# Clinical Conundrums in Management of Hypothyroidism in Critically Ill Geriatric Patients

**DOI:** 10.5812/ijem.13759

**Published:** 2014-01-05

**Authors:** Vishal Sehgal, Sukhminder Jit Singh Bajwa, Rinku Sehgal, Anurag Bajaj

**Affiliations:** 1 The Commonwealth Medical College, Scranton, PA, USA; 2 Department of Anaesthesiology and Intensive Care Medicine, Gian Sagar Medical College, Banur, Patiala, India; 3 The Wright Center for Graduate Medical education, Scranton, PA, USA

**Keywords:** Amiodarone, Kidney Failure, Chronic, Critical Illness, Hypothyroidism, Polypharmacy

## Abstract

**Context:**

Articles in various international and national bibliographic indices were extensively searched with an emphasis on thyroid and hypothyroid disorders, hypothyroidism in elderly hospitalized patients, hypothyroidism in critically ill geriatric population, thyroxine in elderly hypothyroid, drug interactions and thyroid hormones, and thyroid functions in elderly.

**Evidence acquisition:**

Entrez (including PubMed), NIH.gov, Medscape.com, WebMD.com, MedHelp.org, Search Medica, MD consult, yahoo.com, and google.com were searched. Manual search was performed on various textbooks of medicine, critical care, pharmacology, and endocrinology.

**Results:**

Thyroid function tests in elderly hospitalized patients must be interpreted with circumspection. The elderly are often exposed to high iodide content and critical care settings. This may occur because of either decreased iodine excretion or very high intake of iodine. This is especially true for elderly population with underlying acute or chronic kidney diseases or both. Amiodarone, with a very high iodine content, is also often used in this set of population. Moreover, other medications including iodinated contrast are often used in the critical care settings. These may affect different steps of thyroid hormone metabolism, and thereby complicate the interpretation of thyroid function tests.

**Conclusions:**

The current review is aimed at analyzing and managing various clinical aspects of hypothyroidism in hospitalized elderly, and critically ill geriatric patients.

## 1. Context

Thyroid disorders are the second most common endocrine disorders after diabetes mellitus type II (DM-II). Clinical presentation is often vague and nonspecific, and its diagnosis is often dependent on laboratory investigations. Hypothyroidism is sub-classified into three types based on laboratory studies. Primary hypothyroidism is characterized by high thyroid stimulating hormone (TSH) and low free thyroxine (T4) levels. Secondary hypothyroidism is defined as low free T4 and TSH, which is not appropriately increased. Subclinical hypothyroidism (SH) is defined as low normal free T4 and high TSH.

## 2. Evidence Acquisition

Hypothyroidism in geriatric and critically ill elderly patients can be a significant cause for higher morbidity and mortality rates. Hypothyroidism has high prevalence in geriatric population, and is five to eight times more common in women (1, 2). Its incidence is increased with aging. However, the literature estimation of hypothyroidism prevalence among the elderly exhibits a varied picture depending on the studied populations and the criteria to define it. Even in developed nations like the United States (US), the national health and nutrition examination survey (NHANESIII) estimated the prevalence of hypothyroidism approximately 4.6%, (0.3% as overt and 4.3% as SH2). These statistics convey a similar picture among different set-ups and points, with hypothyroid as the second most common endocrine disorder visited by clinicians in geriatric practice (3). Overall, in the US, hypothyroid affects more than 10 million people (4).

Challenges facing clinicians come from the invariably atypical clinical presentation of hypothyroidism. This clinical presentation in hospitalized elderly patients is associated with increased morbidity and mortality rates. The endocrine emergencies exhibit higher morbidity and mortality rates in elderly patients who undergo thyroid surgical procedures (5-8).

## 3. Results 

### 3.1. Literature Search Strategies

Articles in various international and national bibliographic indices were extensively searched with an emphasis on thyroid and hypothyroid disorders, hypothyroidism in elderly hospitalized patients, hypothyroidism in critically ill geriatric population, thyroxine in elderly hypothyroid, drug interactions and thyroid hormones, and thyroid functions in elderly. The emphasis was also on the epidemiology, pattern of clinical presentations, diagnostic modalities, therapeutic interventions and complications due to the hypothyroid disorders in elderly hospitalized patients. Drug-drug interactions as well as the side effects and drug interactions with thyroid hormones were also considered. Entrez (including PubMed), NIH.gov, Medscape.com, WebMD.com, MedHelp.org, Search Medica, MD consult, yahoo.com, and google.com were searched. Manual search was performed on various textbooks of medicine, critical care, pharmacology, and endocrinology.

### 3.2. Hypothyroidism in Hospitalized Elderly: Etiological Factors

According tothe literature, autoimmune thyroiditis is the most common cause of hypothyroidism among the elderly and younger persons. Among other causes, Iodine deficiency, radioiodine ablation, and surgery are significant causes of hypothyroidism in elderly hospitalized patients(9, 10). The incidence of Iodine deficiency related hypothyroidism is decreased in India due to various national initiatives on universal salt iodization (3).

### 3.3. Clinical Epidemiology and Symptomatology of Hypothyroidism in Elderly

Physiological variations between very old and moderately older patients are significant with changes with every passing decade.The symptomatology of hypothyroidism is less specific in elderly patients compared to younger generation, which poses diagnostic challenges to clinicians (Table 1). Moreover, hypothyroidism may often be masked by co-morbidities like DM-II (11). The difficulty in diagnosis is further exaggerated due to various drug interactions related to polypharmacy, which is quite common in this aging population (12). Acute onset of hypothyroidism and thyroid hormone deficiency of higher magnitude can possibly result in exacerbation of symptoms in a robust manner. However, symptoms can be better tolerated if hypothyroidism starts gradually. Both hypothyroidism and SH may be associated with a number of abnormalities which can be present in critically ill patients. These may include but not limited to cardiac, pulmonary, gastro-intestinal (GI), hematological, neurological, and metabolic manifestations (13, 14).

**Table 1.** Common Clinical Presentation of Hypothyroidism in Critically Ill Geriatric Population

Systems	Clinical Manifestations
**Cardiac**	
	Congestive heart failure
	Pericardial effusion
	Hypertension
	Decreased cardiac output
	Angina
**Pulmonary**	
	Decrease response to hypercapnia and hypoxia
	Macroglossia
**Gastrointestinal**	
	Constipation
	Non-alcoholic fatty liver
**Hematologic**	
	Pernicious anemia
	Anemia of chronic disease
**Neurologic**	Complex mental status changes
**Metabolic**	
	Hyperlipidemia
	Hyponatremia
	Metabolic syndrome

Hypothyroidism is a lower risk of atrial fibrillation as compared to a much higher risk in hyperthyroid patients which can directly affect the outcome in critically ill patients with hypothyroidism (15). The resultant reduced cardiac output can contribute to decreased functional cardio-respiratory reserve. Additionally, heart failure and angina may worsen in a patient with acute onset of hypothyroidism and pre-existing coronary artery disease (CAD).However, other cardiac manifestations of hypothyroidism in critical care settings may include but not limited to pericardial effusion, hypertension (HTN) and hyperlipidemia (HLP). It is proven that thyroid hormone replacement therapy (THRT) is beneficial in such critically ill patients (16). Hypothyroidism, both overt and subclinical, are definitely associated with post-prandial HLP; and metabolic syndrome is clarified (17, 18).

Hypothyroidism can also cause weakness of the respiratory muscles and a reduced pulmonary response to hypercapnia and hypoxia. However, shortness of breath seen in hypothyroidism is due to multiple etiologies related to cardiac and pulmonary tissues, while structural abnormalities such as macroglossia may contribute to sleep apnea. These abnormalities could potentially be reversed by appropriate thyroid hormone replacement therapy (THRT) (18).

Constipation is the most common GI complication occurred in hypothyroidism, while frequent association of nonalcoholic fatty liver disease and hypothyroidism is often a matter of clinical concern (19). Among autoimmune disorders, pernicious anemia is associated with 10% of the cases of autoimmune thyroiditis (20). The frequency of anemia of chronic disease is almost equally distributed in both SH and overt hypothyroidism (21).

Central nervous system involvement in hypothyroidism include complex mental status changes, as the main clinical presentation of neurological manifestations(22). Characteristically, Hashimoto’s encephalopathy (HE) which presents as complex mental changes is immune-mediated and not a direct manifestation of hypothyroidism. However, the diagnosis is established only by excluding other causes of mental status changes, also there are some evidences of positive antithyroid antibodies levels. Neurological manifestations of hypothyroidism in critically ill elderly usually present as mental status changes and HE though rare must be in the list of differentials (22). Myxedema coma is an uncommon diagnosis in the ICU, but can be seen when severe hypothyroidism is complicated by infection, trauma, hypothermia narcotics useand benzodiazepines (5, 23). The typical clinical presentation is characterized by a comatose state withpossible association of hyponatremia, hypothermia and hypercapnia.

Hyperthyroidism incidence and levels of antithyroid antibodies increase with aging (24). Due to gradual onset of hyperthyroidism, the classical clinical features of heat intolerance, tachycardia, sweating and tremor may not be seen and are often absent in critically ill patients. Though of limited clinical significance in ICU, subtle signs such as fatigue, muscle weakness, weight loss and atrial arrhythmias are probably present in the patient history (25).

### 3.4. Thyroid Functions in Hospitalized Geriatric Patients

Total or free thyroxine concentrations do not show any marked changes in elderly, because both thyroxine clearance and production decrease with aging (26, 27). At molecular level, such changes are associated with decreased nocturnal pulses of TSH secretion which counterbalances the decreased clearance of thyroxine in the elderly (28). The complicated clinical profile of critically ill geriatric patients mandates a dose reduction and initiation of lower doses of thyroxine supplementation with simultaneous monitoring of TSH levels in spite of wider normal range of TSH in the elderly (29, 30). A number of studies have sought to determine whether biochemical diagnosis of thyroid disorders in the elderly may be confounded by age-related changes in thyroid function. It has also been observed that most elderly people with SH revert to euthyroid state, but higher the TSH levels, more is the possibility of developing overt hypothyroidism (31). TSH within the upper or lower end of normal range is a strong predictor for the possible development of hypo and hyperthyroidism (32). However, low total thyroxine (TT4) and high TSH are strongly associated with poor clinical prognosis in elderly hospitalized patients (33).

### 3.5. Drug Interactions With Thyroid Hormones

**Figure 1.**
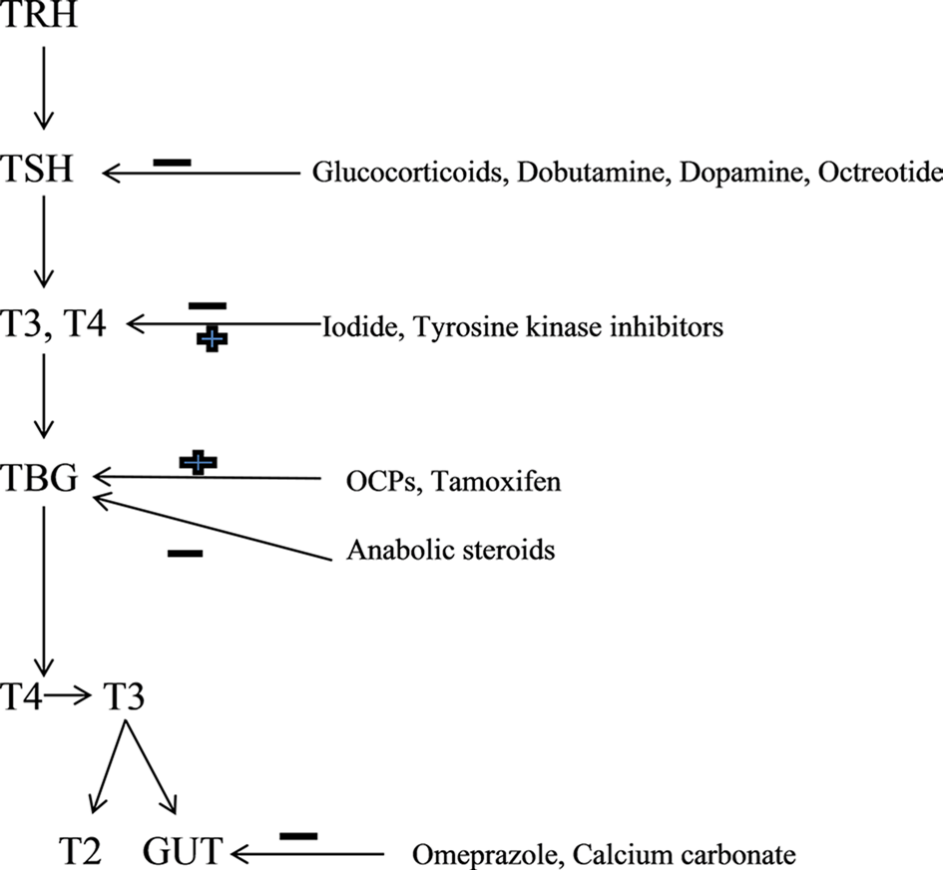
Common Drugs Affecting the Thyroid Hormone Metabolism TRH, Thyrotropin Releasing Hormone; TSH, Thyrotropin; T3, Triiodothyronine; T4, Throxine; T2, Diiodothyronine. Drugs may either inhibit (--) or stimulate (+) various steps in the metabolism of thyroid hormones. Omeprazole inhibits reabsorption of T3 and T4 from the intestine (GUT)

**Figure 2.**
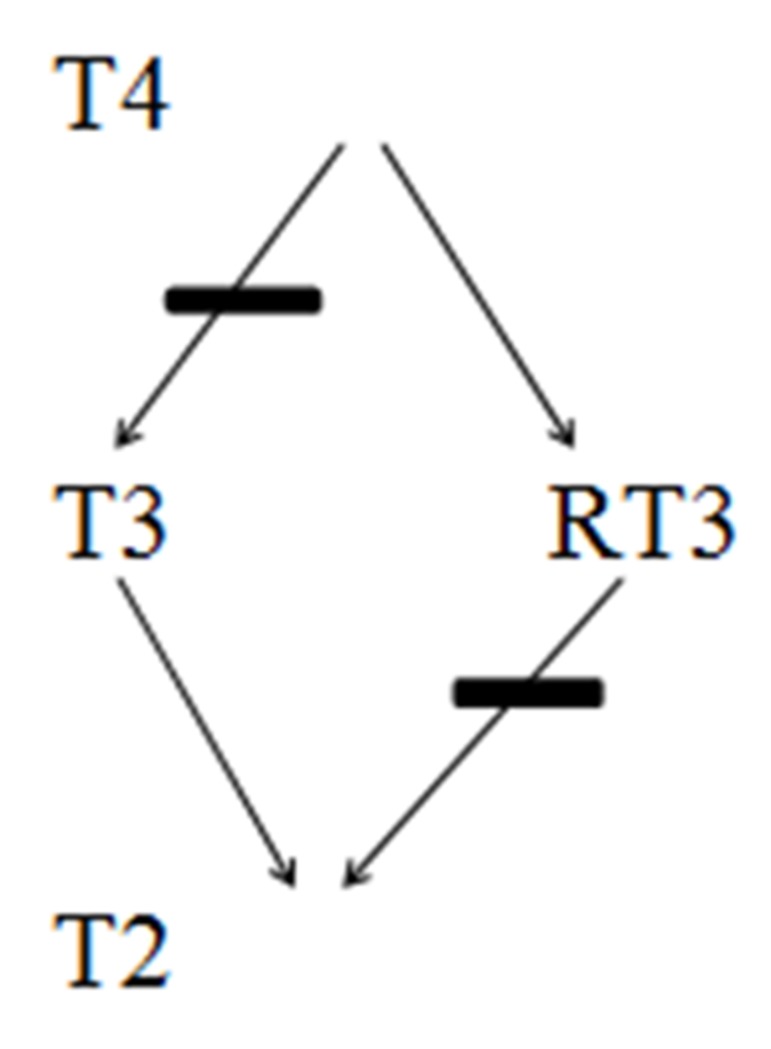
Suppression of T2 and T3 Production by Inhibition of 5-Monodeiodinase Amiodarone, High-dose Steroids, Beta Blockers, Iodinated Contrast, Propylthiouracil, Steroids, Sepsis and NTIS T3, Triiodothyronine; T4,Thyroxine; T2, Diiodothyronine; RT3, Reverse T3

Some of the drugs commonly used in clinical practice may affect the metabolic pathway of thyroid hormones. Drug interactions could potentially affect different steps in the metabolism of thyroid hormones. Elderly usually have multiple co-morbidities and are frequently on polypharmacy (34). This increases the possibility of altered drug metabolism and drug interactions. Depending on the medication class or underlying pathology, drug-induced hyperthyroidism, hypothyroidism or change in thyroid function profile may be seen (Figures 1 and 2).

In critical care setting, drugs that commonly affect TSH secretion may include but not limited to glucocorticoids (more than 20 mg per day of Prednisone or its equivalents), Dobutamine, Dopamine, Octreotide and others. Iodine and Iodine containing drugs such as Amiodarone and contrast agents can potentially cause hyperthyroidism, especially in patients with underlying thyroid adenoma. Wolff-Chaikoff (WC) effect seen in such patients is caused by decreased thyroid hormone synthesis due to decreased oxidation of Iodide resulting from higher doses of Iodine. Antipsychotic medication Lithium is another drug with a potential to cause hypothyroidism. It has also been observed that even therapeutic concentration of Lithium can cause mild hypothyroidism in one-fifth of patients. Development of over thypothyroidism primarily necessitates treatment of hypothyroidism rather than stopping the Lithium therapy. Such patients are likely to need titration of THRT on stoppage of lithium and thus should be closely followed. Tyrosine kinase inhibitors are often used in the treatment of GI and hematological malignancies. Hypothyroidism in such patients with previously normal thyroid isoften preceded by destructive thyroiditis and transient suppression of TSH. Commonly used drugs such as OCPs, Tamoxifen and Methadoneinterfere with thyroid hormone transport and metabolism, and can potentiallyraise TBG. Drugs that affect and interfere with GI absorption of thyroid hormones include bile acid resins, proton pump inhibitors and calcium carbonate (Figure 1).Increased metabolism of T4 and T3 can be potentially influenced by phenobarbital, rifampin, phenytoin and carbamazepine. All these pharmacological interactions necessitate a higher dose of THRT in elderly hospitalized patients. Caution has to be exercised in the usage of drugs such as Amiodarone, steroids and beta blockers in hospitalized elderly as they inhibit 5-monodeiodinase, and thereby cause decreased formation of T3 (Figure 2). 

### 3.6. Thyroid Function Tests in Critical Care Settings

Iodide exposure in critical care settings is one of the key determinants of thyroid hormone dysfunction. Elderly are often exposed to iodide in the critical care settings with increasing use of imaging studies and medications containing high doses of iodide causing impaired iodide auto-regulation mechanisms. The decreased production of thyroid hormones secondary to decreased organification of iodide may lead to WC effect (35). As kidney is largely instrumental in iodide clearance by glomerular filtration, iodide tends to accumulate and may enhance WC effect in patients with renal failure possibly resulting in higher frequency of goiter and hypothyroidism in such patients (36, 37).

Sepsis is commonly encountered in critically ill patients and can increase cytokine levels in the body, which could potentially suppress 5-monodeiodinases (38). The resulting molecular interactions can decrease conversion of T4 to T3 and a decreased release of thyrotrophic releasing hormone (TRH) from the hypothalamus. This clinical presentation is often termed as non-thyroid illness syndrome (NTIS),and also known as sick euthyroid syndrome. This clinical entity is characterized by reverse T3 (RT3) and T4elevation due to inhibition of 5-monodeiodinasesand 5-monodeiodinases, respectively (Figure 2).RT3 may need to be measured to distinguish central hypothyroidism from NTIS, as RT3 levels are high in patients with NTIS except those with renal failure (36). Overall, these endocrinological changes are natural adaptation to reduce the catabolism in the body in diseased states. These lower levels of T3 hormones are highly associated with increased cardiovascular morbidity and mortality rates in uremic patients (39).

Increased incidence of head and neck cancers is also associated with aging. Such cancers are not usually treated with surgical procedures, and may require radiotherapy for therapeutic and palliative purposes. However, a prolonged exposure to radiotherapy can potentially damage the thyroid gland resulting in hypo or hyperthyroidism, which often requires screening when such patients are hospitalized specifically in ICU (40).

### 3.7. Drugs Affecting Thyroid Function in Critical Care Settings

Amiodarone is rich in iodine (250 times the daily recommended dose of iodine),which are commonly prescribed in critical care settings in the elderly population to control atrial fibrillation (AF) (41). This excessively high dose of Iodine has the potential to cause hyperthyroidism or hypothyroidism depending on the clinical scenarios and geographical area of the patient population. In patients living in iodine deficiency areas it causes hyperthyroidism, and in iodine sufficient areas it can cause hypothyroidism (42). Usually during the early phase of therapy, a decrease in T3 and an increase in RT3 and T4 may be observed (Figure 2). T4 and RT3 remain slightly elevated and T3 attains the normal levels after a prolonged period of therapy. A higher incidence of amiodarone-induced hypothyroidism (WC effect) may be observed in patients with underlying autoimmune thyroiditis. On the contrary, Jod-Basedow effect (JB effect) may be observed in patients with underlying goiter or latent Graves’ disease, as the increased availability of iodide substrate can lead to secondary hyperthyroidism. Amiodarone could potentially block 5-monodeiodinasein younger patients. It causes hyperthyroidism in younger patients, while older patients tend to get SH or overt hypothyroidism (43, 44). Most of the patients with no underlying thyroid problems remain euthyroid during amiodarone therapy. However, one study showed that 5 percent of patients developed overt hypothyroidism (TSH > 10 mU/L), and about 25 percent of patients developed SH (TSH 4.5-10) (45). The symptoms of hypothyroidism may be observed even after the treatment with amiodarone has been stopped, which can be explained based on lipophilic nature and its long half-life. As such, a periodic monitoring of TSH in patients treated with amiodarone is essential even in critical care set-ups. 

Dopamine agonists and glucocorticoids commonly used in hospitalized patients may lead to transient central hypothyroidism, and a potential to worsen pre-existing hypothyroidism (46, 47).

### 3.8. Management of Hypothyroidism in Hospitalized Geriatric Patients

Meticulous analysis and scrutiny of drugs and underlying pathology is needed prior to initiating THRT in the inpatient settings. Unwarranted use of THRT in elderly may lead to cardiac arrhythmias with higher chances of developing AF (48). In the critical care settings, geriatric patients may have multivariate clinical presentations with hypothyroidism, hyperthyroidism, SH, NTIS, JB effect, WC effect and HE which can cause diagnostic and therapeutic difficulties.

THRT should be started with caution in elderly, as most of them have underlying CAD. Once cardiovascular tolerance of a starting dose has been assessed, most experts recommend gradually increasing the daily dosage by 12.5-25 microgram (mcg) every four to six weeks. Annual TSH checking may not be necessary in all patients (49).

In non-elderly population, SH treatment leads to better control of cardiac problems (50). In contrast, studies of geriatric populations have not consistently shown similar benefits in treating SH (51). The distribution of TSH among population progressively shifts higher with aging (52). In elderly, there is a higher risk of heart failure, when TSH is higher than 10mU/L (53). THRT may be considered for older people with anti thyroperoxidase antibody and TSH levels more than 10 mU/L (54, 55). SH treatment has been associated with delayed progression of chronic kidney disease in elderly females (56, 57). There is no association between untreated persistent SH and progression of CHF, CAD or mortality, if TSH is less than 10Miu/L in geriatric population (58, 59).TSH levels in these patients may be normalized over the time (60).

NTIS is frequently seen in hospitalized elderly. There is no benefit in treating the same with THRT (61). JB effect is seen in the setting of iodine supplementation with underlying hyperthyroid state, which can be effectively treated by holding the iodine supplements and starting beta-blockers (62). Antithyroid medications may be started to speed up recovery. As JB typically lasts for 1-18 months, TFT should be monitored every 4-6 weeks initially and then every 3 months thereafter. It is usually possible to stop methimazole after 6-12 months of use in hospital.

Iodine induced hypothyroidism is seen in elderly patients with underlying SH. These patients are unusually sensitive to inhibitory effect of iodide, and if there is a need for continued use of these drugs the underlying hypothyroidism should be treated. When HE is suspected, steroids would be needed along with treatment of the dysthyroid state. 

The challenges increase manifold in elderly diabetic and obese patients with associated thyroid disorders (63, 64). A new challenge has been encountered in today’s critical care setting, as many patients with AIDS are admitted with various endocrine disorders(65). Elderly are often on polypharmacy and thyroid medications may be lost during medication reconciliation in transition from oral to enteral route in the ICU (66). Multidisciplinary team approach would help in ensuring appropriate and cost effective treatment in critically ill geriatric patients with hypothyroidism.
